# Dan-Lou Prescription Inhibits Foam Cell Formation Induced by ox-LDL via the TLR4/NF-κB and PPARγ Signaling Pathways

**DOI:** 10.3389/fphys.2018.00590

**Published:** 2018-05-29

**Authors:** Li-Na Gao, Xin Zhou, Yu-Ren Lu, Kefeng Li, Shan Gao, Chun-Quan Yu, Yuan-Lu Cui

**Affiliations:** ^1^Tianjin University of Traditional Chinese Medicine, Tianjin, China; ^2^College of Pharmacy, Jining Medical University, Rizhao, China; ^3^Research Center of Traditional Chinese Medicine, Tianjin University of Traditional Chinese Medicine, Tianjin, China; ^4^Key Research Laboratory of Prescription Compatibility among Components, Tianjin University of Traditional Chinese Medicine, Tianjin, China; ^5^Tianjin Sunnypeak Biotech Co., Ltd., Tianjin, China

**Keywords:** Dan-Lou prescription, oxidative low-density lipoprotein (ox-LDL), TLR4, NF-κB, PPARγ

## Abstract

Atherosclerosis is the major worldwide cause of mortality for patients with coronary heart disease. Many traditional Chinese medicine compound prescriptions for atherosclerosis treatment have been tried in patients. Dan-Lou prescription, which is improved from Gualou-Xiebai-Banxia decoction, has been used to treat chest discomfort (coronary atherosclerosis) for approximately 2,000 years in China. Although the anti-inflammatory activities of Dan-Lou prescription have been proposed previously, the mechanism remains to be explored. Based on the interaction between inflammation and atherosclerosis, we further investigated the effect of Dan-Lou prescription on macrophage-derived foam cell formation and disclosed the underlying mechanisms. In the oxidative low-density lipoprotein (ox-LDL) induced foam cells model using murine macrophage RAW 264.7 cells, the ethanol extract from Dan-Lou prescription (EEDL) reduced ox-LDL uptake and lipid deposition by inhibiting the protein and mRNA expression of Toll-like receptor (TLR)4 and scavenger receptor (SR)B1. After stimulation with ox-LDL, the metabolic profile of macrophages was also changed, while the intervention of the EEDL mainly regulated the metabolism of isovalerylcarnitine, arachidonic acid, cholesterol, aspartic acid, arginine, lysine, L-glutamine and phosphatidylethanolamine (36:3), which participated in the regulation of the inflammatory response, lipid accumulation and cell apoptosis. In total, 27 inflammation-related gene targets were screened, and the biological mechanisms, pathways and biological functions of the EEDL on macrophage-derived foam cells were systemically analyzed by Ingenuity Pathway Analysis system (IPA). After verification, we found that EEDL alleviated ox-LDL induced macrophage foam cell formation by antagonizing the mRNA and protein over-expression of PPARγ, blocking the phosphorylation of IKKα/β, IκBα and NF-κB p65 and maintaining the expression balance between Bax and Bcl-2. In conclusion, we provided evidences that Dan-Lou prescription effectively attenuated macrophage foam cell formation via the TLR4/NF-κB and PPARγ signaling pathways.

## Introduction

Atherosclerosis is the major cause of mortality for patients with coronary heart disease worldwide. It is characterized by complex interactions, including lipid deposition, vascular smooth muscle cell proliferation, endothelial dysfunction and extracellular matrix remodeling (Lim and Park, 2014). Since the 1800s, the concept of atherosclerosis related to inflammation has been reported (Wong et al., 2012). Until the 2000s, more and more researchers have appreciate that inflammatory mechanisms couple dyslipidemia to atheroma formation (Libby, 2002). The “inflammation hypothesis” is further strongly acknowledged combining the detection of circulating inflammatory markers in patients and possibility of selective gene modification in mice (Libby et al., 2002). The therapeutic strategies for attenuating inflammation have been the focus of multiple clinical trials and most anti-inflammatory therapies used in phase II and III trials show promising results (Al-Hawwas and Tanguay, 2013).

When lipoproteins are modified in the arterial wall, the immune response is activated, and then, monocyte-derived cells differentiate into macrophages to ingest the accumulated lipoproteins (Moore et al., 2013). After ingesting modified lipoprotein, such as oxidized LDL (ox-LDL), the macrophages are transformed into cholesterol-loaded foam cells. The foamy appearance of the macrophage cytoplasm alters the phenotype, immune function, and migratory capacity and is regarded as an inducer of inflammation deterioration in atherosclerosis (Montero-Vega, 2014). The uptake of ox-LDL can be recognized by SRs including SRA, SRB1 and CD36 (Moore and Tabas, 2011). After CD36 recognizes ox-LDL in association with Toll-like receptor (TLR)s, nuclear factor (NF)-κB is activated and the inflammatory cascade is triggered (Choudhury et al., 2005), deteriorating the initiation and progression of atherosclerosis (Sun et al., 2014). TLR4 signaling has been focused as a potential therapeutic target in ischaemic coronary artery disease (Jia et al., 2014). Various drugs exert anti-atherosclerosis effects, such as statins and thiazolidinediones, via targeting TLR4. Moreover, some traditional Chinese herbs, which have not been applied for the treatment of coronary heart disease, have been proven to be beneficial for the prevention of atherosclerosis. For example, rhubarb exerts an anti-atherosclerotic effect due to anti-inflammatory activities mediated by TLR4 (Zhang et al., 2013). In atherosclerosis, ox-LDL binding to CD36 results in a positive feedback loop via activation of peroxisome proliferator activated receptor gamma (PPARγ) (Lim et al., 2006). PPARγ activation by ox-LDL is related to protein kinase B (PKB), PKC and mitogen-activated protein kinase (MAPK). It has been reported that curcumin blocks ox-LDL-induced foam cell formation in RAW 264.7 cells by inhibiting the expression of CD36 and PPARγ (Min et al., 2013).

It should be noted that macrophages govern the inception, progression and terminal manifestation of the inflammatory response in atherosclerosis (Moore and Tabas, 2011). Various macrophage subtypes are activated by different stimuli within the plaque. Especially, neutrophils and monocyte-derived macrophages participate in early plaque progression (lesion initiation), advanced plaque progression and inflammatory cascade aggravation (Moore and Tabas, 2011; Nahrendorf and Swirski, 2015). The outbreak of atherosclerosis is the result of a fatty streak, which is composed almost entirely of monocyte-derived macrophages (Hartman and Frishman, 2014). As the atheroma develops, T cells, mast cells and other inflammatory cells are also recruited into the intima (Moore and Tabas, 2011). Next, the second critical feature, the necrotic core, triggers plaques breakdown and induces stroke, sudden heart death, and myocardial infarction. In atherosclerosis, the balance of macrophages in the plaque is dynamic (Moore et al., 2013). Responding to different environmental signals, plaque-associated macrophages express pro- and anti-atherogenic factors to influence lipid metabolism, inflammatory responses and plaque stability (Mantovani et al., 2009).

One of the attractive therapeutics for atherosclerosis is to switch a pro-inflammatory phenotype to an anti-inflammatory phenotype (Al-Hawwas and Tanguay, 2013). In the clinic, hydroxymethyl glutaryl coenzyme A reductase inhibitors (statins) have been proven as effective drugs in decreasing low-density lipoprotein (LDL) cholesterol levels in cardiovascular events (Al-Hawwas and Tanguay, 2013). In addition, statins are regarded as anti-inflammatory agents. Many traditional anti-inflammatory agents targeting varied pathways have been tried in patients with the symptoms of atherosclerosis.

**FIGURE 1:**
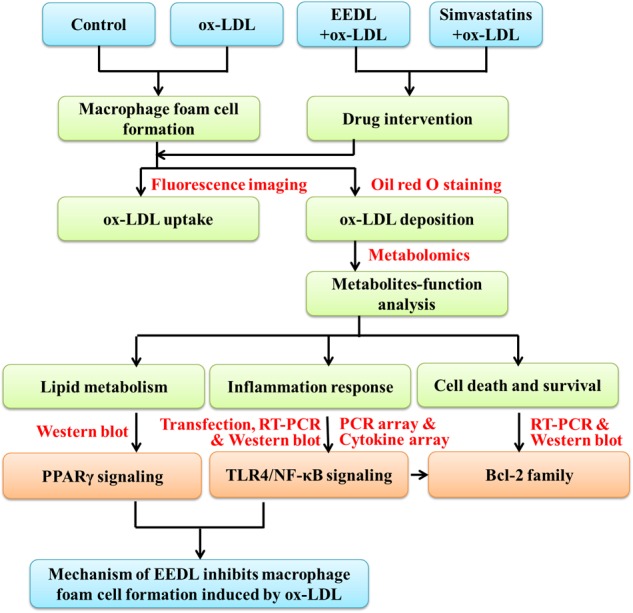
The schematic diagram of the study.

Dan-Lou prescription, which is administrated to patients with chest discomfort, is derived from the traditional Chinese medicine compound prescription Gualou-Xiebai-Banxia decoction. Previously, we have prepared the EEDL and identified the eight highest level ingredients gallic acid, salvianic acid, puerarin, daidzin, paeoniflorin, salvianolic acid B, cryptotanshinone and tanshinone IIA (Gao et al., 2015). Although the anti-inflammatory effect has preliminarily been proposed as one of the potential mechanisms of the EEDL for coronary heart diseases treatment, the underlying mechanisms remain unclear. Macrophage-derived foam cell formation is the important warning sign of atherosclerosis. In this study, we attempted to declare the underlying mechanism of the EEDL in macrophage-derived foam cells formation induced by ox-LDL in murine macrophage RAW 264.7 cells, based on the interaction between inflammation and atherosclerosis (**Figure 1**). SEM imaging, Oil Red O staining and 1,1^′^-dioctadecyl-3,3,3^′^,3^′^-tetramethylindocyanide percholorate (DiI) labeled ox-LDL uptake were combined to show the cell morphology, lipid accumulation, and ox-LDL uptake. A systematic metabolomics, PCR array, cytokine array and network pharmacological analyses were combined to screen targeting molecules and explore signaling pathways with EEDL intervention. Moreover, the regulation of PPARγ and TLR/NF-κB signaling pathways were verified by real-time reverse-transcription polymerase chain reaction (RT-PCR) and Western blotting.

## Materials and Methods

### Reagents

The drug powder of Dan-Lou prescription was provided by Jilin Connell Medicine Co., Ltd. (Jilin, China). The EEDL was prepared as previously described by us (Gao et al., 2015). DMEM-high glucose, Oil Red O and 3-(4,5-dimethylthiazol-2-yl)-2,5-diphenyltetrazolium bromide (MTT) and were obtained from Sigma–Aldrich Co. (St. Louis, MO, United States). Heat inactivated fetal bovine serum (HI-FBS) was purchased from Biological Industries (Kibbutz Beit-Haemek, Israel). Ox-LDL and DiI labeled ox-LDL (DiI-ox-LDL) were purchased from Yiyuan Biotechnologies (Guangzhou, China). Mammalian Cell Lysis Kit, UNIQ-10 column Trizol total RNA extraction kit, and antibodies for LOX-1, SRB1 and β-actin were obtained from Sangon Biological Engineering Technology & Services Co., Ltd. (Shanghai, China). The TNF-α Mouse ELISA kit and MCP-1 Mouse ELISA kit were obtained from Peprotech (Rocky Hill, CT, United States). The antibody for PPARγ was purchased from proteintech (Chicago, IL, United States). The antibody for COX-2 was obtained from BD Pharmingen (San Diego, CA, United States) and goat anti-mouse IgG peroxidase conjugate was purchased from Merck (Darmstadt, Germany). Antibodies for TLR4, phospho-IKKα/β (P-IKKα/β), P-IκBα and P-NF-κB p65 were purchased from Cell Signaling Technology (Beverly, MA, United States). Antibodies for Bcl-2 and Bax were obtained from Beijing Zhongshan Jinqiao Biotechnology Co., Ltd. (Beijing, China). The Improm-II Reverse Transcription System was purchased from Promega Corporation (Madison, WI, United States). The FastStart Universal SYBR Green Master (ROX) kit was purchased from Roche (Mannheim, Germany). The RT^2^ First Strand Kit and RT^2^ SYBR Green Master Mix were obtained from QIAGEN (Hilden, Germany). BAY 11-7082 was obtained from Beyotime Institute of Biotechnology (Haimen, China). Lipofectamine 2000 was obtained from Life Technologies (Carlsbad, CA, United States). The NF-κB Ready-To-Glow Secreted Luciferase Reporter Assay Kit was purchased from Clontech Laboratories, Inc. (Mountain View, CA, United States). The Mouse Cytokine Array Panel A Array kit was obtained from R&D systems (Minneapolis, MN, United States).

### Cells and Cell Culture

The murine macrophage cell line RAW 264.7 was obtained from Cell Culture Center of Chinese Academy of Medical Sciences (Beijing, China). Cells were cultured in medium (DMEM supplemented with 10% HI-FBS, 100 U/mL penicillin and 100 μg/mL streptomycin) at 37°C in a fully humidified incubator containing 5% CO_2_. For all experiments, cells were grown to a confluence of 80–90%, and were subjected to no more than seven cell passages. Different cell densities were cultured for the MTT assay (1.0 × 10^4^ cells per well), SEM imaging (1 × 10^5^ cells per well), Oil Red O staining (4 × 10^4^ cells per well), ELISA (1 × 10^5^ cells per well), real-time reverse-transcription polymerase chain reaction (RT-PCR), PCR array, cytokine array, Western blotting and metabolomics (1.5 × 10^6^ cells per well). Next, cells were treated with medium (5% HI-FBS without phenol), EEDL (400, 200, and 100 μg/mL) or simvastatin (10 μM) in the presence or absence of ox-LDL (100 μg/mL) for another 24 or 48 h for determination. In all experiments, macrophages in the control group were treated medium only without EEDL and ox-LDL.

### Cell Viability Assay

To investigate the effect of the EEDL, ox-LDL and simvastatin on RAW 264.7 cells, we firstly screened the concentrations without cytotoxicity using the MTT assay, which is based on the enzyme conversion of MTT in mitochondria (Mosmann, 1983). Briefly, 0.5 mg/mL of MTT was added and incubated for 4 h at 37°C. The resulting formazan crystals were dissolved in DMSO and the absorbance was recorded at 490 nm using an EnSpire Multimode Plate Reader (PerkinElmer, United States).

### Cell Micrograph Analysis

Ox-LDL stimulation promotes the macrophages to transform into cholesterol-loaded foam cells with the alteration of phenotype, immune function and migratory capacity. In this study, we observed the cell morphology by SEM imaging. RAW 264.7 cells were washed twice with PBS, and were fixed with 4% paraformaldehyde for 15 min at room temperature. Next, the cells were dehydrated through graded solutions of ethanol (30, 50, 70, 90, and 100%) twice with an incubation of 15 min and subjected to 100% acetonitrile. Finally, samples were prepared by vacuum drying, coated with gold and examined with a SEM (Superscan SS-550, Shimadzu, Japan). The number and diameter of cells were quantitatively analyzed using NIH ImageJ software^1^ (NIH, United States) and the analyst was blinded to the treatment group.

### Oil Red O Staining

Oil Red O staining is a classical method to monitor foam cell formation in macrophages from various origins such as monocyte/macrophage cell lines and bone marrow-derived macrophages (Xu et al., 2010). For Oil Red O staining, RAW 264.7 cells were fixed with 4% paraformaldehyde for 15 min. After washed with PBS, the cells were stained with Oil Red O (dissolved in isopropanol with a final concentration of 0.3 mg/mL) for 30 min. Subsequently, the cells were washed with PBS three times to remove background staining. Finally, lipid formation in intracellular droplets was observed under microscope (Olympus, Japan). Simultaneously, to quantify the extent of foam cells formation, lipid accumulated in the cells was extracted with isopropanol and the absorbance was recorded at 530 nm using an EnSpire Multimode Plate Reader (PerkinElmer, United States).

### Fluorescence Imaging of ox-LDL Uptake

To examine cellular ox-LDL uptake, RAW 264.7 cells (5 × 10^4^ cells/well) were cultured overnight in 35-mm dishes. Next, the cells were pre-incubated with medium (without phenol and, with 5% HI-FBS), EEDL (400 μg/mL) or simvastatin (10 μM) in the presence or absence of ox-LDL (100 μg/mL) for 44 h. Next, the culture medium was changed to that containing DiI-ox-LDL (40 μg/mL), followed by incubation for another 4 h to test the specificity of uptake. For nuclear staining, the cells were incubated with DAPI (0.3 μg/mL) for 5 min. Finally, the cells were washed and examined by confocal laser scanning microscope (Zeiss LSM 710, Germany).

### Cellular Metabolite Extraction and Metabolomics Profiling

Cellular metabolites can reflect the physiological and pathological states of cells. In this study, metabolomics was used to explore the change in the metabolites before and after EEDL intervention in ox-LDL-induced RAW 264.7 cells. In addition to the control group, cells were treated with medium (without phenol, added 5% HI-FBS) or EEDL (400 μg/mL) with ox-LDL (100 μg/mL) for another 24 h, and the RAW 264.7 cells were washed with pre-chilled PBS three times and scraped with 0.5 mL of pre-chilled extract solution (methanol: ddH_2_O = 21:79, v/v) into 2-mL centrifuge tubes. Next, 1 mL of CHCl_3_ was added to the suspension. The samples were sonicated three times (3 s per time), incubated for 15 min on ice for metabolite extraction, and centrifuged at 12,000 ×*g* for 15 min at 4°C. The supernatants were collected, dried under nitrogen, and, finally, re-extracted with 0.1 mL of mobile phase for LC-MS/MS analysis.

The maintain metabolites were measured by the LC-MS/MS system and comprised a Shimadzu LC-20AD Qtrap 5500 tandem mass spectrum (SCIEX, United States). Briefly, two injections were conducted, one on the positive mode and the other one on the negative mode according to a previous study with modifications (Yuan et al., 2012). Ten microliters of the respective extracts were injected by a PAL CTC autosampler into a 150 × 2 mm, 4 μm apHera NH_2_ high-performance liquid chromatography (HPLC) column (Supelco, United States) held at 25°C for chromatographic separation. The mobile phase consisted of A (95% ddH_2_O + 5% acetonitrile + 20 μM ammonium hydroxide, pH 9.4) and B (100% acetonitrile). The flow rate was set at 0.5 mL/min. The elution was carried out as 0–3 min, 95% B; 3–6 min, 75% B; 6–7 min, 0% B; 7–12 min, 0% B, and 12–15 min, 95% B. The mass spectrometer via the electrospray source was operated in both the positive ion (5500 V)/ and negative ion (-4500 V) modes under scheduled multiple reaction monitoring conditions (MRM). The switch time was set at 50 ms. The temperature was 500°C. In total, 420 metabolites were targeted.

Metabolomics data were log2-transformed. The PLS-DA, metabolic pathways and volcano plots were constructed using the Metaboanalyst platform^2^. Metabolites with variable importance in the projection (VIP) scores greater than 1.5 were considered as significant.

### PCR Array and Protein Array Analyses

The effect of the EEDL on the TLR signaling pathway in ox-LDL induced macrophage foam cells was detected by RT^2^ Profile PCR Array (QIAGEN, Germany). In addition to the control group, RAW 264.7 cells were treated with medium (without phenol, added 5% HI-FBS) or EEDL (400 μg/mL) in the presence of ox-LDL (100 μg/mL) for 24 h. Cells were washed with pre-chilled PBS twice, and total RNA was extracted using the UNIQ-10 column Trizol total RNA extraction kit (Sangon, China) following the commercial instructions. Thereafter, cDNA was synthesized as described by the RT^2^ First Strand Kit instructions, and equivalent cDNA was mixed with the RT^2^ SYBR Green Master Mix for each profiling plate using ABI 7500 Real-time PCR system (Applied Biosystems, United States). Data analysis used the ΔΔC_T_ method^3^.

The Mouse Cytokine Array Panel A Array kit (R&D, United States) was used according to the manufacturer’s instructions to screen the targeting cytokines regulated by EEDL in ox-LDL-induced macrophage foam cells. The procedure of cells treatment was similar to that of PCR array. After cells were washed with pre-chilled PBS twice, protein was extracted using a Mammalian Cell Lysis Kit (Sangon, China) and was quantified by the BCA method (Pierce, United States). In total, 20 μg of protein was run on the array. After the array was scanned into a computer and saved as image files, images were semi-quantified using NIH ImageJ software.

### Pathway and Network Analysis by IPA

Twenty-seven gene targets were obtained from PCR array and cytokine profile array analyses. The targeted inflammatory-related genes, chemokines and cytokines were uploaded into the IPA^4^ to explore the molecular interaction and identify the biological mechanisms, pathways and biological functions of the EEDL in ox-LDL-induced macrophage foam cell formation.

### ELISA for TNF-α, MCP-1 and COX-2

To detect the tumor necrosis factor (TNF)-α and monocyte chemoattractant protein (MCP)-1 levels, cell supernatants were collected and analyzed using commercial ELISA kits (Peprotech, United States) according to the manufacturer’s instructions. To determine cyclooxygenase (COX)-2 protein expression, cells were fixed with 4% paraformaldehyde, and a modified original protocol for the cell-based ELISA was applied (Versteeg et al., 2000; Gao et al., 2013). Finally, cells were incubated with 0.2% Janus Green B to calibrate the differences in cell number (Raspotnig et al., 1999).

### Real-Time RT-PCR and Western Blot Analyses

Total RNA extract and protein purification were performed simultaneously. Briefly, the cells were washed with pre-chilled PBS twice and mRNA was extracted using the Sangon UNIQ-10 column Trizol total extraction kit (Sangon, China). Reverse transcriptions was performed following the instructions of the ImProm-II Reverse Transcription System cDNA synthesis kit (Promega, United States). The real-time RT-PCR oligonucleotide primers used for mouse PPARγ, LOX-1, SRB1, TLR4, TNF-α, MCP-1, COX-2, Bax, Bcl-2 and β-actin are shown in **Table 1**.

**Table 1:** The real-time RT-PCR oligonucleotide primers.

Gene	Primer	Sequence (5^′^-3^′^)	PCR product (bp)
β-actin	Forward	TGTTACCAACTGGGACGACA	165
(NM_007393.3)	Reverse	GGGGTGTTGAAGGTCTCAAA	
COX-2	Forward	TGAGTACCGCAAACGCTTCTC	151
(NM_011198.3)	Reverse	TGGACGAGGTTTTTCCACCAG	
MCP-1	Forward	CCCAATGAGTAGGCTGGAGA	125
(NM_011333.3)	Reverse	TCTGGACCCATTCCTTCTTG	
TNF-α	Forward	TAGCCAGGAGGGAGAACAGA	127
(NM_013693.2)	Reverse	TTTTCTGGAGGGAGATGTGG	
Bax	Forward	CTGCAGAGGATGATTGCTGA	174
(NM_007527.3)	Reverse	GATCAGCTCGGGCACTTTAG	
Bcl-2	Forward	GGACTTGAAGTGCCATTGGT	127
(NM_177410.2)	Reverse	AGCCCCTCTGTGACAGCTTA	
SRB1	Forward	GGGCTCGATATTGATGGAG	171
PPARγ	Forward	GATGGAAGACCACTCGCATT	115
(NM_001127330.2)	Reverse	AACCATTGGGTCAGCTCTTG	
(NM_016741.2)	Reverse	GGAAGCATGTCTGGGAGGTA	
TLR4	Forward	GGCAGCAGGTGGAATTGTAT	198
(NM_021297.3)	Reverse	AGGCCCCAGAGTTTTGTTCT	
LOX-1	Forward	TGGTGGATCCAGATGTTTGA	99
(NM_001301096.1)	Reverse	GTTGGTTGGGAGACTTTGGA	


After RNA isolation, protein was precipitated with isopropyl alcohol for 10 min, followed by centrifugation at 12,000 ×*g* for 10 min. Next, guanidine hydrochloride (0.3 M) was added to denature the protein for 20 min. The protein precipitation was obtained by centrifugation at 7,500 ×*g* for 5 min. Thereafter, the protein prescription was washed with ethanol. After, aspirating the ethanol, 1% SDS was added to dissolve the protein at 50°C. The protein concentration was determined using BCA assay (Pierce, United States). Equal amounts (20 μg) of protein lysate were separated by SDS-PAGE and boiled for 5 min. Subsequently, the samples were transferred to polyvinylidene difluoride (PVDF) membranes, blocked with TTBS (0.5% Tween 20, 10 mM Tris-HCl, pH 7.5, 150 mM NaCl) containing 5% non-fat milk for 1 h at room temperature and incubated with antibodies against PPARγ (1:2000), LOX-1, TLR4, SRB1, P-IKKα/β, P-IκBα and P-NF-κB p65, Bcl-2 and Bax (1:1000) or β-actin (1:5000) overnight at 4°C. The membranes were washed and further incubated with HRP-conjugated secondary antibodies against mouse (1:1000 for Bax and Bcl-2, and 1:10000 for β-actin) or rabbit (1:1000 for LOX-1, P-IKKα/β, P-IκBα and P-NF-κB p65, 1:5000 for PPARγ, TLR4 and SRB1) for 1 h at room temperature. After washing, the protein bands were detected using an ECL detection kit (Millipore, United States) and exposure to Kodak BioMax Light films. The films were scanned into a computer and semi-quantified by NIH ImageJ software.

### Transient Cell Transfection and Luciferase Reporter Assay

Activation of NF-κB was monitored using a pNF-κB-MetLuc2 reporter (Clontech, United States) that, contains an NF-κB enhancer element upstream of a secreted luciferase (MetLuc) gene. RAW 264.7 cells were plated in 48-well plates (2.5 × 10^5^ cells per well) overnight. Transfection of cells was performed using Lipofectamine 2000 (Life, United States) according to the manufacturer’s instructions. After transfection for 24 h, the cells were incubated with medium (without phenol, added 5% HI-FBS), EEDL (400, 200, and 100 μg/mL) or BAY 11-7082 (10 μM) for 2 h and then treated with ox-LDL (100 μg/mL). After 3 h, 50 μL of culture media were transferred from each sample into a 96-well plate. Next, 5 μL of substrate buffer was added and luciferase activity was recorded using an EnSpire Multimode Plate Reader (PerkinElmer, United States).

### Statistical Analysis

Origin 8.0 software (MicroCal, United States) was applied to perform the statistical analysis. N indicated the number of wells studied in each category. The data were expressed as the means ± SD. For statistical comparisons, the results were analyzed using one-way analysis of variance (ANOVA) and *P* < 0.05 was considered statistically significant.

## Results

### Cell Viability Evaluation

The effect of ox-LDL, EEDL and simvastatin on RAW 264.7 cells was detected using the MTT assay (**Supplementary Figure S1**). Compared with the control group, the EEDL (800 μg/mL) inhibited the cell proliferation (48.02% for EEDL-treated alone group and 46.92% for EEDL+ox-LDL group). However, no significant variation in the optical density was observed among other groups. Additionally, no inhibition was observed in the ox-LDL and simvastatin groups. For further study, we aimed to investigate how EEDL protected cells from ox-LDL-induced macrophage foam cell formation, and to elucidate the underlying mechanism. To avoid the direct effect of EEDL on cell viability, the concentrations of EEDL chosen were 400, 200, and 100 μg/mL.

### EEDL Restores Cell Morphology and Suppresses Lipid Deposition

**FIGURE 2:**
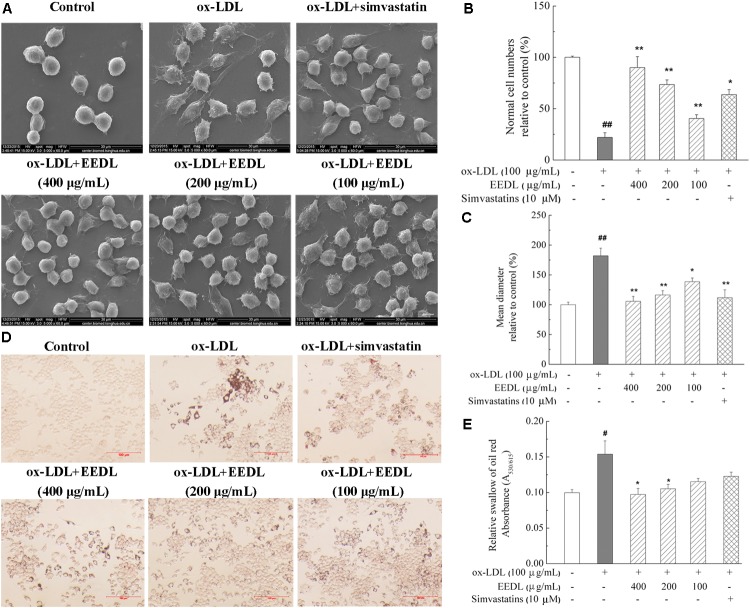
Cellular morphology and representative photomicrographs of lipid-laden RAW 264.7 cells. **(A)** Representative images of SEM. Analysis of normal cell numbers **(B)** and mean diameter **(C)** of cells. The numbers of normal cells per field (1 mm^2^) and mean diameter of 50 cells randomly chosen were quantitatively analyzed using NIH ImageJ software (*n* = 4). **(D)** Oil Red O staining. The scale bar was 100 μm. **(E)** Lipid accumulation in cells. Lipid accumulation in intracellular droplets was extracted with isopropanol and the absorbance was recorded at 530 nm (*n* = 6). Significance compared with the control group or ox-LDL treated alone, ^#^*P* < 0.05, ^##^*P* < 0.01 vs. the control group, ^∗∗^*P* < 0.01 or ^∗^*P* < 0.05 vs. the ox-LDL-treated group.

Macrophage-derived foam cells play a vital role in the initiation and development of atherosclerosis (Moore and Tabas, 2011). Thus, it is indispensable to efficiently suppress macrophage foam cell formation for atherosclerosis treatment. SEM micrographs (**Figure 2A**) displayed that ox-LDL stimulated RAW 264.7 cells to become large, and the antennae increased. In the control group, the cells were characterized by a smooth surface, good refraction and fewer pseudopodia and were defined as “normal cells.” Compared with the control group, ox-LDL decreased the normal cells iper field (1 mm^2^) to 19.23% (**Figure 2B**, *P <* 0.01). Similarly, ox-LDL increased the mean diameter of cells to 182.5% (**Figure 2C**, *P <* 0.01), compared with that of the control group. However, the EEDL and simvastatin significantly down-regulated the tendency of ox-LDL to induce abnormal cell morphology (*P <* 0.05 or *P <* 0.01).

We further determined the accumulation of lipid droplets in ox-LDL induced RAW 264.7 cells. As shown in **Figure 2D**, after stimulation with ox-LDL for 48 h, the Oil Red O-stained lipid droplets were distributed throughout the cytosol of most cells, while no lipid droplet were observed in the control group. With the intervention of EEDL or simvastatin, intracellular lipid droplets were markedly reduced. For the EEDL treatment, the optical density detection also displayed a decrease in ox-LDL-induced lipid accumulation (**Figure 2E**, *P* < 0.05).

### EEDL Suppresses ox-LDL Uptake of Macrophage-Derived Foam Cells

**FIGURE 3:**
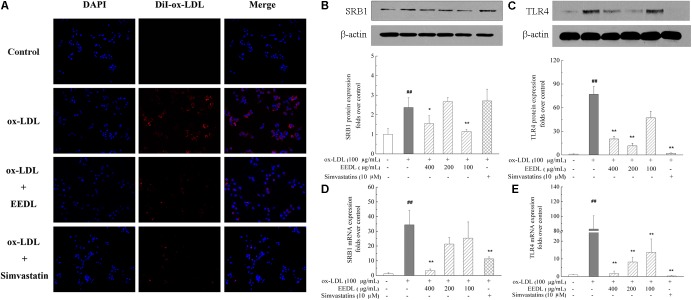
Effect of the EEDL on ox-LDL uptake in RAW 264.7 cells. **(A)** DiI-ox-LDL uptake in RAW 264.7 cells. Besides the control group, cells were incubated in the presence of DiI-ox-LDL (40 μg/mL) for 4 h. After washed and fixed, cell nucleus was stained with DAPI. DiI-ox-LDL uptake was shown in red and nucleus was shown in blue. Determination of SRB1 **(B)** and TLR4 **(C)** protein expression was detected by Western blotting. The relative optical density was quantified using NIH ImageJ software. Values are means ± SD (*n* = 3). The mRNA expression of SRB1 **(D)** and TLR4 **(E)** was detected using real-time RT-PCR. Values are means ± SD (*n* = 3) from three independent experiments. Significance compared with the control group or ox-LDL treated alone, ^##^*P* < 0.01 vs. the control group, ^∗∗^*P* < 0.01, ^∗^*P* < 0.05 vs. the ox-LDL-treated group.

The fluorescent dyes that are routinely used to investigate foam cells formation are Nile Red and DiI. To visualize the uptake of ox-LDL, in this study, fluorescent DiI-ox-LDL was applied. When DiI-ox-LDL accumulated in RAW 264.7 cells increasingly, it indicated the process of ox-LDL uptake. When excess ox-LDL was bound or internalized to RAW 264.7 cells, the macrophages were transformed into foam cells. As illustrated in **Figure 3A**, ox-LDL greatly promoted cellular uptake of DiI-ox-LDL. In the process of DiI-ox-LDL uptake, EEDL or simvastatin markedly inhibited the binding and internalization of DiI-ox-LDL in RAW 264.7 cells. Furthermore, we screened receptor-mediated ox-LDL uptake. As shown in **Figures 3B–E**, the mRNA and protein expression levels of SRB1 and TLR4 were increased by ox-LDL induction. Compared with that of the ox-LDL group, tshe EEDL reduced both the mRNA and protein expression levels of SRB1 and TLR4 (*P <* 0.05 or *P <* 0.01). Simultaneously, we detected the expression of LOX-1. As shown in **Supplementary Figure S2**, EEDL showed no significant inhibition in the up-regulation of LOX-1 induced by ox-LDL.

### EEDL Modifies the Metabolic Profile of ox-LDL-Induced Macrophage-Derived Foam Cells

**FIGURE 4:**
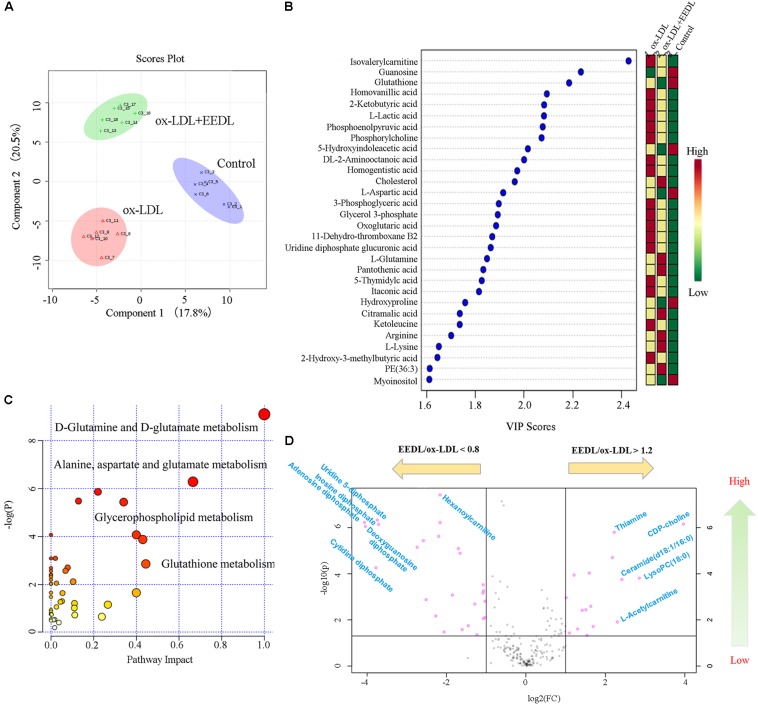
Modification of the EEDL on metabolites in ox-LDL-induced macrophage foam cells. **(A)** PLS-DA analysis among control, ox-LDL and ox-LDL + EEDL groups. **(B)** The top 30 differential metabolites among control, ox-LDL and ox-LDL + EEDL groups by VIP analysis. **(C)** The differential metabolic pathways altered after EEDL intervention. **(D)** Vocano plot analysis between ox-LDL and ox-LDL + EEDL groups.

Metabolomics is considered as an important tool in the diagnosis and prognosis of CVD because it can help interpret the pathogenesis of CVD (Ussher et al., 2016). Measurement of ox-LDL metabolites such as malondialdehyde and conjugated diene has been proven to be important for children with a high family risk for premature CVD (Kelishadi et al., 2002). In this study, we investigated the metabolic profile of the EEDL treatment in ox-LDL induced macrophage foam cells. As displayed in **Figure 4A**, the control, ox-LDL and ox-LDL+EEDL groups were completely separated by multivariate partial least-square discriminant analysis (PLS-DA), indicating that the intervention of EEDL significantly altered the metabolites of ox-LDL-induced macrophage foam cells. The top 30 most different cellular metabolites among the control, ox-LDL and ox-LDL + EEDL groups are listed in **Figure 4B**, and mainly included carnitine (isovalerylcarnitine), nucleotides (guranosine, 5-thymidylic acid), amino acids (aspartic acid, arginine, lysine, glutamine), cholesterol, and phosphatidylethanolamine (PE, 36:3). Pathway analysis (**Figure 4C**) showed that these different metabolites belong to D-glutamine and D-glutamate metabolism, alanine, aspartate and glutamate metabolism, glycerophospholipid metabolism and glutathione metabolism pathways. Compared with the ox-LDL group, volcano plot analysis (**Figure 4D**) showed that EEDL increased the content of ceramide, thiamine, and CDP-choline, but reduced the level of hexanoylcarnitine and cytidine diphosphate. Ceramide exists in the cell membrane, and participates in the regulation of cell immunology, proliferation, differentiation and apoptosis (Chalfant and Spiegel, 2005). Mechanisms of thiamine deficiency have been focused on oxidative stress, inflammation and excitotoxicity (Hazell and Butterworth, 2009). CDP-choline is the middle product for lecithin biosynthesis. Peripheral administration of CDP-choline can alleviate acute inflammatory pain (Gurun et al., 2009). The increase in ceramide, thiamine, and CDP-choline indicated that EEDL may antagonize ox-LDL-induced foam cell formation through anti-inflammatory activities.

### Targeting Genes and Proteins Screening

**FIGURE 5:**
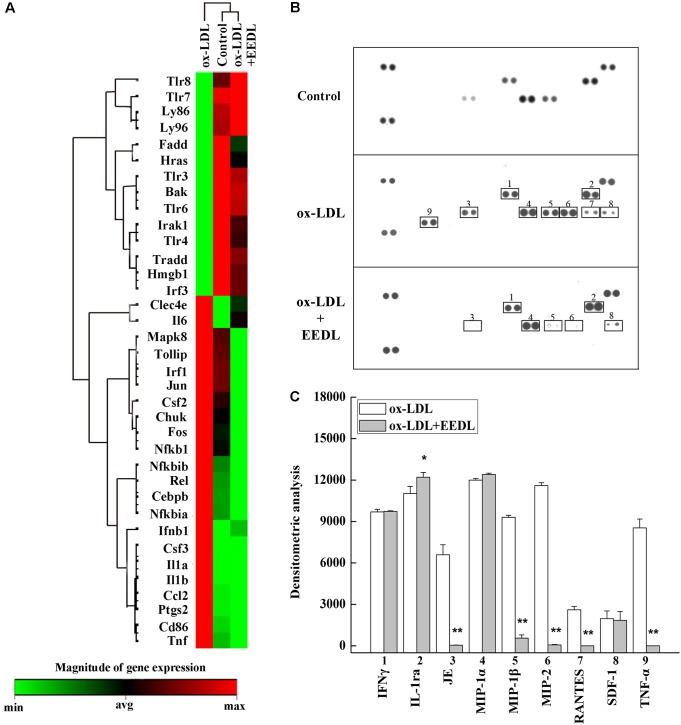
Targets screened based on PCR array and protein array analyses. **(A)** The mRNA expression of the EEDL antagonized macrophage-derived foam cell formation was evaluated using PCR array based on TLR signaling pathway. **(B)** Cytokines and chemokines inactivated by the EEDL in the ox-LDL-induced macrophage-derived foam cells were screened by the Mouse Cytokine Array Panel A Array system. **(C)** Analysis of optical density was captured by the NIH ImageJ software. ^∗∗^*P* < 0.01, ^∗^*P* < 0.05 vs. the ox-LDL-treated group.

The TLR signaling pathway, mediated by relevant COX-2, NF-κB and peroxidase directly affect the metabolism of RAW 264.7 cells (Li and Glass, 2002). Combined with the metabolic profile abovementioned, we used PCR to screen TLR signaling pathway-related targeting genes of EEDL that antagonize ox-LDL-induced foam cell formation. Significant changes in the mRNA expression pattern induced by ox-LDL were shown in **Figure 5A**. After EEDL treatment, the gene expression was close to that of the control group, indicating that EEDL reversed ox-LDL induced dysregulation of gene expression. The targeting genes screened (**Table 2**) were *Ccl-2 (MCP-1)*, *Csf2*, *Csf3*, *IL-1a*, *IL-1b*, *NF-κB*, *Ptgs2*, and *Tnf*, which belong to the NF-κB pathway, JAK/STAT pathway and cytokine-mediated signaling pathway.

**Table 2:** The mRNA expression regulated by EEDL in ox-LDL induced macrophage foam cells via Toll-like receptor signaling pathway (EEDL vs. ox-LDL).

Gene symbol	Fold regulation	Gene symbol	Fold regulation
Ccl2	-73.99	Il1b	-810.73
Cd86	-14.18	Jun	-2.60
Cebpb	-3.10	Nfkb1	-2.35
Csf2	-125.42	Nfkbia	-5.05
Csf3	-386.78	Nfkbib	-2.66
Fos	-2.72	Ptgs2	-174.54
Ifnb1	-3.84	Rel	-4.22
Il1a	-77.05	Tnf	-5.69


The role of inflammation in atherosclerosis has received extensive attention. The use of anti-inflammatory or immunomodulatory therapies for atherosclerosis has been shown to be effective in clinical studies (Al-Hawwas and Tanguay, 2013). Next, a Mouse Cytokine Array Panel A Array kit was used to confirm the inflammatory cytokines regulated by EEDL. As shown in **Figures 5B,C**, the targeting cytokines were IL-1ra, JE (MCP-1), MIP-1β, MIP-2, RANTES and TNF-α. Cytokines are important regulatory factors in the inflammatory cascade, mediating leukocyte migration and invasion, and regulating cell growth and differentiation in atherosclerosis, which play a vital roles in the initial stage (von Hundelshausen and Schmitt, 2014; Zernecke and Weber, 2014). In this study, the EEDL selectively inhibited the over-expression of MCP-1, MIP-1β, MIP-2 and TNF-α (*P* < 0.01), enhancing the secretion of IL-1ra (*P* < 0.05).

### Network Pharmacology Analysis

**FIGURE 6:**
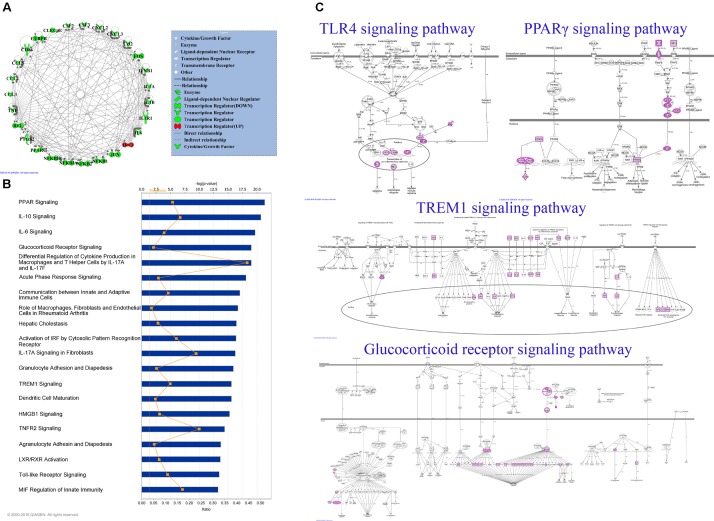
Network analysis by IPA software. **(A)** Network interaction among targeting genes and proteins obtained from PCR array and protein array. In total, 27 molecules were uploaded to analyze the molecule interaction. Green color indicated down-regulation and red indicated up-regulation. **(B)** The top 20 enrichment pathways of the EEDL on ox-LDL induced macrophage foam cell formation. **(C)** The detailed TLR4, PPARγ, TREM1 and glucocorticoid receptor signaling pathways.

Based on the results of PCR array and protein array, we used IPA to analyze the molecular interaction, signal transduction pathways, and biological functions. The interaction of different targets is shown in **Figure 6A**. The top 20 signal transduction pathways of EEDL to inhibit macrophage foam injury are shown in **Figure 6B**, including the glucocorticoid receptor, TREM1, PPARγ, and TLR4. The targeting molecular and main signaling pathways are shown in detail in **Figure 6C**. The top 15 biological functions are shown in **Table 3**, including cell death and survival, inflammatory response, cell-to-cell signaling and cellular function.

**Table 3:** Biological function categories of EEDL inhibited macrophage foam cell formation by IPA software (top15).

Categories	Diseases or Functions Annotation	*p*-value	Molecules
Cell-to-cell signaling and interaction, cellular movement	Recruitment of cells	1.83E-30	21
Cell death and survival	Apoptosis of leukocytes	2.94E-30	22
Cellular movement, immune cell trafficking	Leukocyte migration	2.59E-29	25
Inflammatory response	Inflammation of absolute anatomical region	1.58E-28	26
Gene expression	Binding of protein binding site	1.6E-28	19
Cell death and survival	Cell death of immune cells	1.75E-28	23
Cellular development, cellular growth and proliferation	Proliferation of blood cells	1.65E-27	24
Hematological system development and function, tissue morphology	Quantity of blood cells	1.77E-27	25
Cellular function and maintenance, hematological system development and function	Function of myeloid cells	3.28E-27	17
Inflammatory response, organismal injury and abnormalities	Inflammation of organ	1E-26	26
Cellular function and maintenance, hematological system development and function	Function of macrophages	3.47E-26	16
Cellular movement	Cellular infiltration	3.83E-26	20
Cell-to-cell signaling and interaction	Activation of cells	4.68E-26	24
Cellular function and maintenance	Function of phagocytes	8.21E-25	17
Cell-to-cell signaling and interaction, hematological system development and function, immune cell trafficking	Adhesion of immune cells	1.53E-24	18


### EEDL Inactivates the PPARγ and NF-κB Signaling Pathways

**FIGURE 7:**
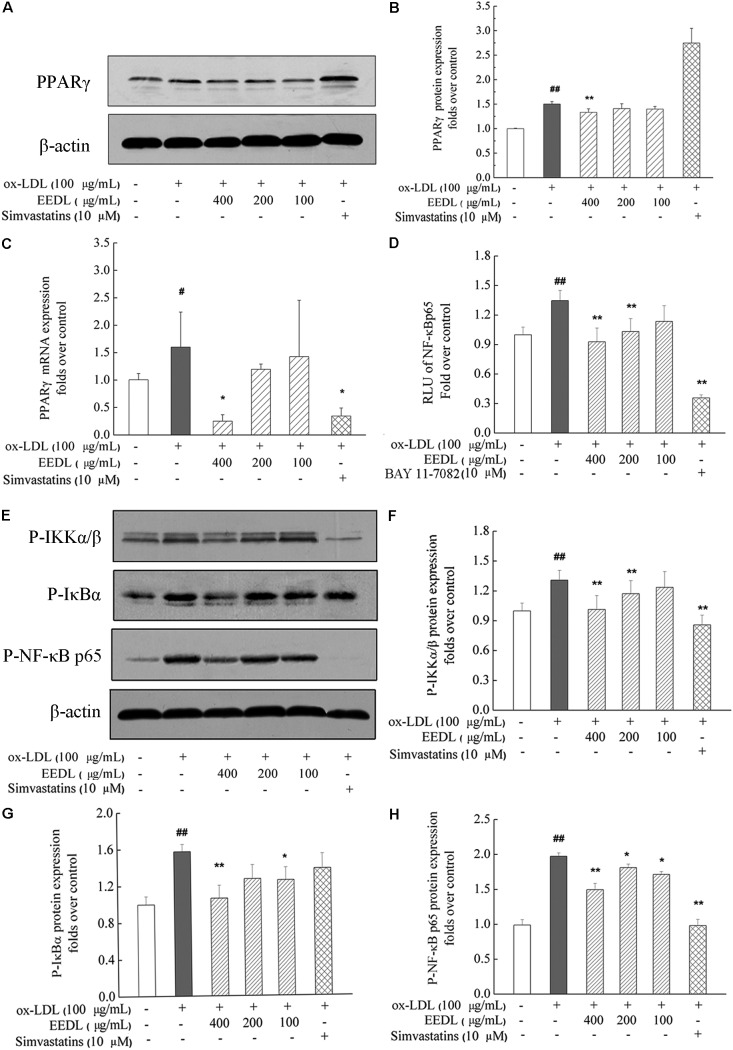
Inactivation of the PPARγ and NF-κB in ox-LDL-induced macrophage foam cell formation. **(A,B)** The protein expression of PPARγ. **(C)** The mRNA expression of PPARγ. **(D)** Inactivation of the NF-κB in transfected RAW 264.7 cells using luciferase reporter, pNF-κB-MetLuc2. **(E)** Western blotting results of P-IKKα/β, P-IκBα and P-NF-κB p65. The relative optical density analysis of P-IKKα/β **(F)** P-IκBα **(G)** and P-NF-κB p65 **(H)**. The relative optical density of protein bands were quantified using NIH ImageJ software. Values are means ± SD (*n* = 3). Significant difference compared with the control group or ox-LDL treated alone, ^#^*P* < 0.05, ^##^*P* < 0.01 vs. the control group, ^∗∗^*P* < 0.01, ^∗^*P* < 0.05 vs. the ox-LDL-treated group. The protein expression of P-NF-κB p65, P-IκBα, and P-IKKα/β was detected in the same gel in which β-actin was used as the internal control.

As similar to the IPA analysis, EEDL significantly decreased ox-LDL induced over-expression of PPARγ from both protein and mRNA levels (**Figures 7A–C**, *P* < 0.05 or *P* < 0.01). NF-κB controls the transcription of many genes with an established role in atherosclerosis (De Winther et al., 2005). Whether EEDL influenced the ox-LDL induced NF-κB signaling pathway activation, RAW 264.7 cells were transfected with a pNF-κB-MetLuc2 reporter. Twenty-six hours later, samples from different groups were withdrawn and luciferase activity was measured. We found that ox-LDL remarkably promoted the activation of NF-κB (**Figure 7D**, *P* < 0.01). However, EEDL and the NF-κB inhibitor, BAY 11-7082, reversed the activation induced by ox-LDL (*P* < 0.01). Next, the protein expression of P-IKKα/β, P-IκBα, and P-NF-κB p65 were measured by Western blotting. The EEDL and simvastatin are shown to block the ox-LDL-stimulated phosphorylation of IKKα/β, IκBα, and NF-κB p65 (**Figures 7E–H**, *P* < 0.05 or *P* < 0.01).

**FIGURE 8:**
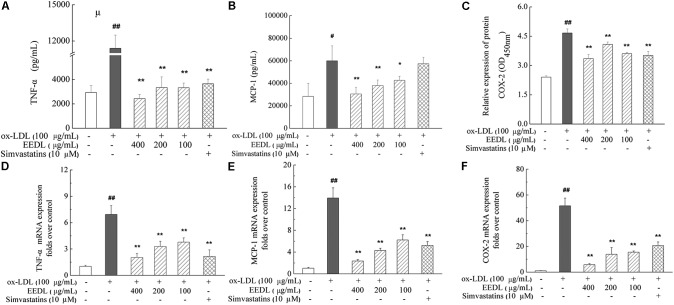
Regulation of the TNF-α, MCP-1 and COX-2 expression in ox-LDL-induced RAW 264.7 cells. The protein expression of TNF-α **(A)**, MCP-1 **(B)** and COX-2 **(C)** was determined using ELISA and cell-based ELISA. Effect of the EEDL on the mRNA expression level of TNF-α **(D)**, MCP-1 **(E)** and COX-2 **(F)** was detected using real-time RT-PCR. Significant difference compared with the control group or ox-LDL treated alone, ^##^*P* < 0.01, ^#^*P* < 0.05 vs. the control group, ^∗∗^*P* < 0.01, ^∗^*P* < 0.05 vs. the ox-LDL-treated group.

When NF-κB is activated, it is free to translocate to the nucleus in which it facilitates the transcription of cytokines and chemokines. The decrease in MCP-1 was associated with suppression of other inflammatory markers, including TNF-α and COX-2, indicating inactivation of the NF-κB signaling pathway. In ox-LDL-induced RAW 264.7 cells, ox-LDL was demonstrated to significantly increase the mRNA and protein expression levels of TNF-α, MCP-1 and COX-2 (**Figure 8**, *P* < 0.05 or *P* < 0.01). After intervention with the EEDL or simvastatin, an aberrant increase in mRNA and protein expression levels of TNF-α, MCP-1 and COX-2 was decreased (*P* < 0.05 or *P* < 0.01).

### EEDL Restores the Abnormal Expression of Bax and Bcl-2

**FIGURE 9:**
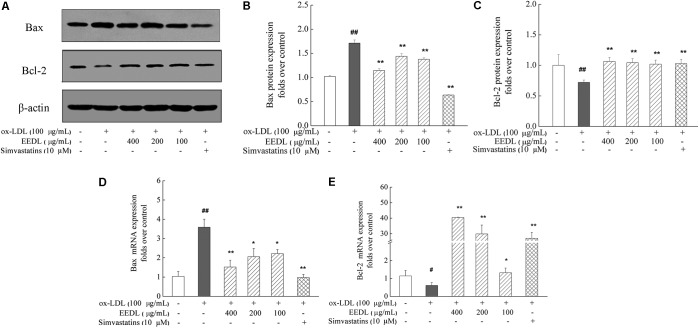
Regulation of the Bax and Bcl-2 expression in ox-LDL-induced RAW 264.7 cells. **(A)** Effect of the EEDL and simvastatin on ox-LDL-induced protein expression of Bax and Bcl-2 was detected using Western blotting. The relative optical density analysis of the Bax **(B)** and Bcl-2 **(C)** was quantified using NIH ImageJ software. Values are means ± SD (*n* = 3). The mRNA expression of Bax **(D)** and Bcl-2 **(E)** was detected using real-time RT-PCR. Values are means ± SD (*n* = 3) from three independent experiments. Significance compared with the control group or ox-LDL treated alone, ^##^*P* < 0.01, ^#^*P* < 0.05 vs. the control group, ^∗∗^*P* < 0.01, ^∗^*P* < 0.05 vs. the ox-LDL-treated group. The protein expression of the Bax and Bcl-2 was detected in the same gel in which β-actin was used as internal control.

Studies have reported that the pro-apoptotic effect of ox-LDL play a key role in atherosclerosis on human monocytes isolated from peripheral blood (PBMs), the U937 monocyte cell line and human monocyte-derived macrophages (Ermak et al., 2008). In this study, we investigated the anti-apoptotic effect of the EEDL on ox-LDL induced macrophage foam cells. Ox-LDL stimulation showed an increase in the mRNA and protein levels of Bax and simultaneously inhibited the mRNA and protein expression of Bcl-2 (**Figure 9**, *P* < 0.05 or *P* < 0.01). The EEDL intervention markedly decreased the expression of Bax and increase the expression of Bcl-2 in both the mRNA and protein levels (*P* < 0.05 or *P* < 0.01). Simvastatin also antagonized the up-regulation of Bax and down-regulation of Bcl-2 induced by ox-LDL (*P* < 0.01).

## Discussion

Inflammation is a key player throughout atherosclerotic plaque development, and many researchers have focused on screening inflammatory-related biomarkers for immunomodulatory treatments (Siasos et al., 2015; Sorci-Thomas and Thomas, 2016). Although robust evidences have supported the use of anti-inflammatory treatment strategies for atherosclerosis treatment, more clinical trials should be performed to provide additional insights into atherosclerosis. Translational research, connecting basic research technologies with clinical practice, may be a better way to reach the ultimate goal (Lauer and Skarlatos, 2010).

Dan-Lou prescription is improved from Gualou-Xiebai-Banxia decoction and has been used for approximately 2,000 years in China. In the clinic, it is mainly used for CVD, such as angina pectoris, acute myocardial infarction (Wang et al., 2015). In our previous study, we determined the main chemical components of the EEDL by HPLC and proposed that the EEDL is a potent anti-inflammatory agent by inhibiting iNOS/NO, COX-2/prostaglandin (PG)E_2_ and cytokine expression (Gao et al., 2015). To better clarify the active chemical components of Dan-Lou prescription regarding their pharmacological effects, we combined chemical biology and network pharmacology to elucidate the cardiovascular disease specificity and mechanisms of action. We found that puerarin (with the highest content in the EEDL) is the main component to attenuate atherosclerosis by regulating leptin and the LDL receptor (Wang et al., 2015). The interaction between ox-LDL and macrophages plays an important role in the plaque initiating and promoting processes. Ox-LDL influences cell apoptosis and proliferation of macrophages. In this study, we focused on the effect of the EEDL on ox-LDL induced foam cells formation and tried to disclose the mechanisms.

Enzymes, such as MPO, iNOS and NADPH oxidases, have been confirmed to participate in LDL oxidation in human atherosclerosis lesions (Li and Glass, 2002). Macrophages can express the abovementioned enzymes and amplify the oxidative reactions to worsen atherosclerotic lesions (Li and Glass, 2002). Macrophages digest ox-LDL abnormally, resulting in lipid accumulation and foam cells formation. From the view of cell morphology, in this study, we found that the EEDL maintained a more normal cell appearance with the stimulation of ox-LDL. The EEDL antagonized the bond and internalization of ox-LDL and decreased ox-LDL induced lipid accumulation in RAW 264.7 cells. The mechanism of ox-LDL uptake has been widely investigated. It is noteworthy that TLR4, SRs such as SRA1, SRB1 and lectin-like ox-LDL receptor (LOX)-1 act as pattern recognition molecules of ox-LDL (Ichimura et al., 2008). They are important in macrophage-derived foam cell formation by binding and internalizing oxidized lipids, accelerating the deterioration of atherosclerosis (Zhang et al., 2014). In this study, we proposed that the decreased uptake of ox-LDL by the EEDL was related to the down-regulation of SRB1 and TLR4.

Metabolomics has also been increasingly applied in cell lines for pharmacological effects under various conditions (Leon et al., 2013). Macrophages have been regarded as a major source of nearly all the lipid mediators (Rouzer et al., 2006; Zai et al., 2007). In this study, several endogenous chemical mediators of the EEDL were screened, and included ceramide, arachidonic acid, carnitine, phospholipids, L-glutamine and amino acids. The abovementioned metabolites participated in the processes of cell apoptosis (Zhou et al., 1998), the inflammatory response (Davies et al., 1984), energy metabolism, lipid metabolism and oxidative stress.

TLR4 is another portal for macrophages to activate downstream cascades in atherosclerosis lesions. TLR4 provides a potential link between inflammation and lipids deposition (Xu et al., 2001). TLR4/TLR6 or SR/TLR cooperates with CD36 to activate NF-κB and amplify the pro-inflammatory responses in response to ox-LDL (Moore et al., 2013). In this study, we combined the TLR signaling pathway PCR array and cytokine protein array to screen inflammatory-related targeting molecules from the EEDL on macrophage-derived foam cells. The main targeting molecules included MCP-1, PGE_2_, TNF-α, MIP and CSF. IPA analysis showed that the relevant signaling pathways mainly included PPARγ, TLR4 and inflammatory-related signaling pathways.

PPARs regulate the expression of many genes, involving in metabolism, inflammation, apoptosis and oxidative stress. Ox-LDL stimulation can increase the expression of PPARγ and CD36 via the activation of the p38 MAPK signaling pathway (Min et al., 2013). In this study, we also found that ox-LDL increased the expression of PPARγ in RAW 264.7 cells. Furthermore, EEDL inhibited the expression of PPARγ from both mRNA and protein levels. The deeply mechanism should be explored because activation of PPARγ also mediates cholesterol efflux, while the RAW 264.7 cells model is not appropriate. We proposed that the PPARγ regulated cholesterol metabolism should be investigated *in vivo*.

NF-κB participates in the processes of apoptosis and foam cells formation. The inhibition of IKKβ attenuates inflammatory injury and lipid deposition in macrophages, which has potential for atherosclerosis therapy (Jacobs, 2006). Apoptosis in macrophages is triggered by cooperation between TLR4 and macrophage pattern recognition receptors (such as SRA1 and LOX-1) (Isner et al., 1995). Studies in transgenic mice have shown that proteins in the Bcl-2 family are main regulators in cell death relevant to atherosclerosis (Westra, 2010). The deterioration of atherosclerosis lesions is mainly due to macrophage death. To investigate the anti-apoptotic action of the EEDL, in this study, we detected the effect of the EEDL on the expression of Bcl-2 and Bax from both the mRNA and protein levels. The results showed that the EEDL exerted an anti-apoptotic effect by inhibiting the expression of Bax and enhancing the expression of Bcl-2, indicating that the anti-apoptotic effect of the EEDL may be one of the mechanisms to prevent atherosclerosis.

## Conclusion

**FIGURE 10:**
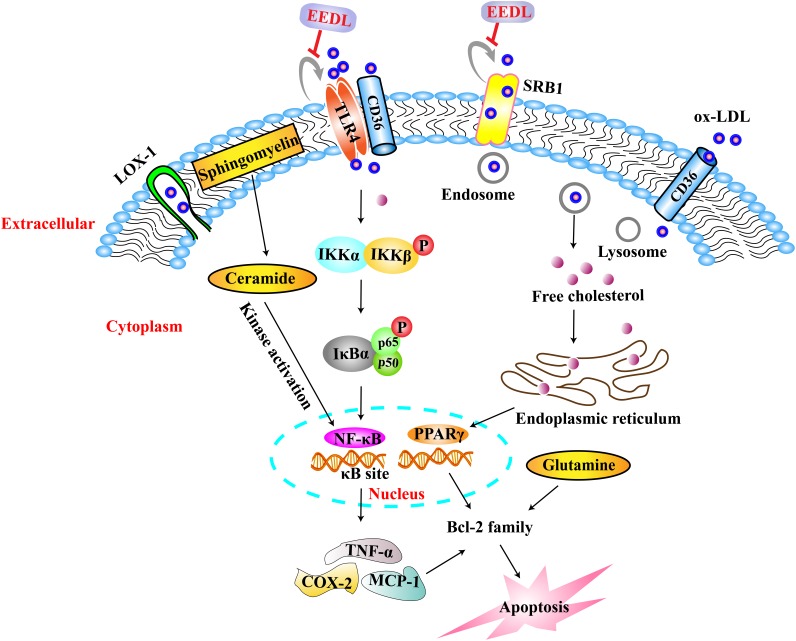
Mechanisms of EEDL on ox-LDL-induced foam cell formation in RAW 264.7 cells. Ox-LDL entered into macrophages via LOX-1, TLR4, SRB1 and CD36, and induced lipid accumulation via PPARγ signaling pathway. Then the content of cell metabolites was changed such as cholesterol, ceramide, L-glutamine and arachidonic acid, resulting in lipid deposition, inflammatory damage, energy metabolism dysfunction, cell apoptosis. However, the EEDL mainly regulated TLR4 and SRB1 to inhibit the uptake of ox-LDL, inhibited PPARγ expression, and following, restored the abnormal metabolites induced by ox-LDL. In the TLR4/NF-κB signaling pathway, the EEDL mainly blocked the phosphorylation of IKKα/β, IκBα and NF-κB p65. Thereafter, the EEDL decreased the expression of TNF-α, COX-2 and MCP-1 to exert anti-inflammatory effect, and inhibited apoptosis in macrophages via regulating Bcl-2 family. In conclusion, EEDL prevented macrophages foam cell formation via the TLR4/NF-κB and PPARγ signaling pathways.

Dan-Lou prescription effectively attenuated macrophage foam cell formation via multiple signaling pathways, including TLR4/NF-κB and PPARγ. The targeting molecules are shown in **Figure 10**, and include TLR4, PPARγ, NF-κB, MCP-1, COX-2, TNF-α, Bcl-2 and Bax. The targeted metabolites included ceramide, glutamine, and arachidonic acid. We also hypothesized that Dan-Lou prescription regulated the crosstalk among inflammation, lipid deposition and apoptosis. The research will be continued in future work, in which the correlations between the components and pharmacological effects may be declared.

## Author Contributions

L-NG drafted and reviewed the manuscript, performed the PCR array, protein array, metabolomics, network pharmacology analyses, and statistical analysis. XZ performed the Western blot, real-time RT-PCR, SEM and cell transfection experiments. Y-RL performed the network pharmacology analysis. Y-LC conceived, designed, and supervised the study and revised the manuscript. KL contributed metabolomics analysis. SG provided guidance to the study. C-QY designed and supervised the study. All authors read and approved the manuscript.

## Conflict of Interest Statement

The authors declare that the research was conducted in the absence of any commercial or financial relationships that could be construed as a potential conflict of interest.

## Footnotes

1 http://rsb.info.nih.gov/ij/

2 www.metaboanalyst.ca">http://www.metaboanalyst.ca">www.metaboanalyst.ca

3 http://www.qiagen.com/shop/genes-and-pathways/data-analysis-center-overview-page/

4 http://www.ingenuity.com

## Supplementary Material

FIGURE S1 Cell viability detection. Cells viability was measured using MTT assay. Values are means ± SD (*n* = 6) from three independent experiments. Significance compared with the control group ^##^*P* < 0.01.

FIGURE S2 Effect of the EEDL on LOX-1 expression in ox-LDL-induced RAW 264.7 cells. **(A)** The protein expression of LOX-1 was detected using Western blotting. The relative optical density was quantified using NIH ImageJ software. Values are means ± SD (*n* = 3). **(B)** The mRNA expression of LOX-1 was detected using real-time RT-PCR. Values are means ± SD (*n* = 3) from three independent experiments. Significance compared with the control group or ox-LDL treated alone, ^##^*P* < 0.01 vs. the control group, ^∗∗^*P* < 0.01 vs. the ox-LDL-treated group.

## Abbreviations

COX-2: cyclooxygenase 2
CVD: cardiovascular diseases
DiI-ox-LDL: 1,1^′^-dioctadecyl-3,3,3^′^,3^′^-retramethylindocyanide percholorate (DiI)-labeled oxidative low-density lipoprotein
DMEM: Dulbecco’s modified Eagle’s medium
EEDL: ethanol extract from Dan-Lou prescription
ELISA: enzyme-linked immunosorbent assay
HI-FBS: heat-inactive fetal bovine serum
HPLC: high performance liquid chromatography
iNOS: inducible nitric oxide synthase
IPA: Ingenuity Pathway Analysis system
LOX-1: lectin-like ox-LDL receptor 1
MCP-1: monocyte chemoattractant protein 1
MPO: myeloperoxidase
MTT: 3-(4, 5- dimethylthiazol-2-yl)-2, 5-diphenyltetrazolium bromide
NF-κB: nuclear factor κB
PGE_2_: prostaglandin E_2_
PPARγ: peroxisome proliferator activated receptor γ
RT-PCR: real-time reverse transcription polymerase chain reaction
SDS: sodium dodecyl sulfate
SEM: scanning electron microscope
SR: scavenger receptor
TLR4: Toll-like receptor 4
TNF-α: tumor necrosis factor α
