# Supplementary figures and images for: Dan-Lou Prescription Inhibits Foam Cell Formation Induced by ox-LDL via the TLR4/NF-κB and PPARγ Signaling Pathways
Source: Front Physiol. 2018 May 29;9:590. doi: 10.3389/fphys.2018.00590 (PMC5987004; doi:10.3389/fphys.2018.00590)

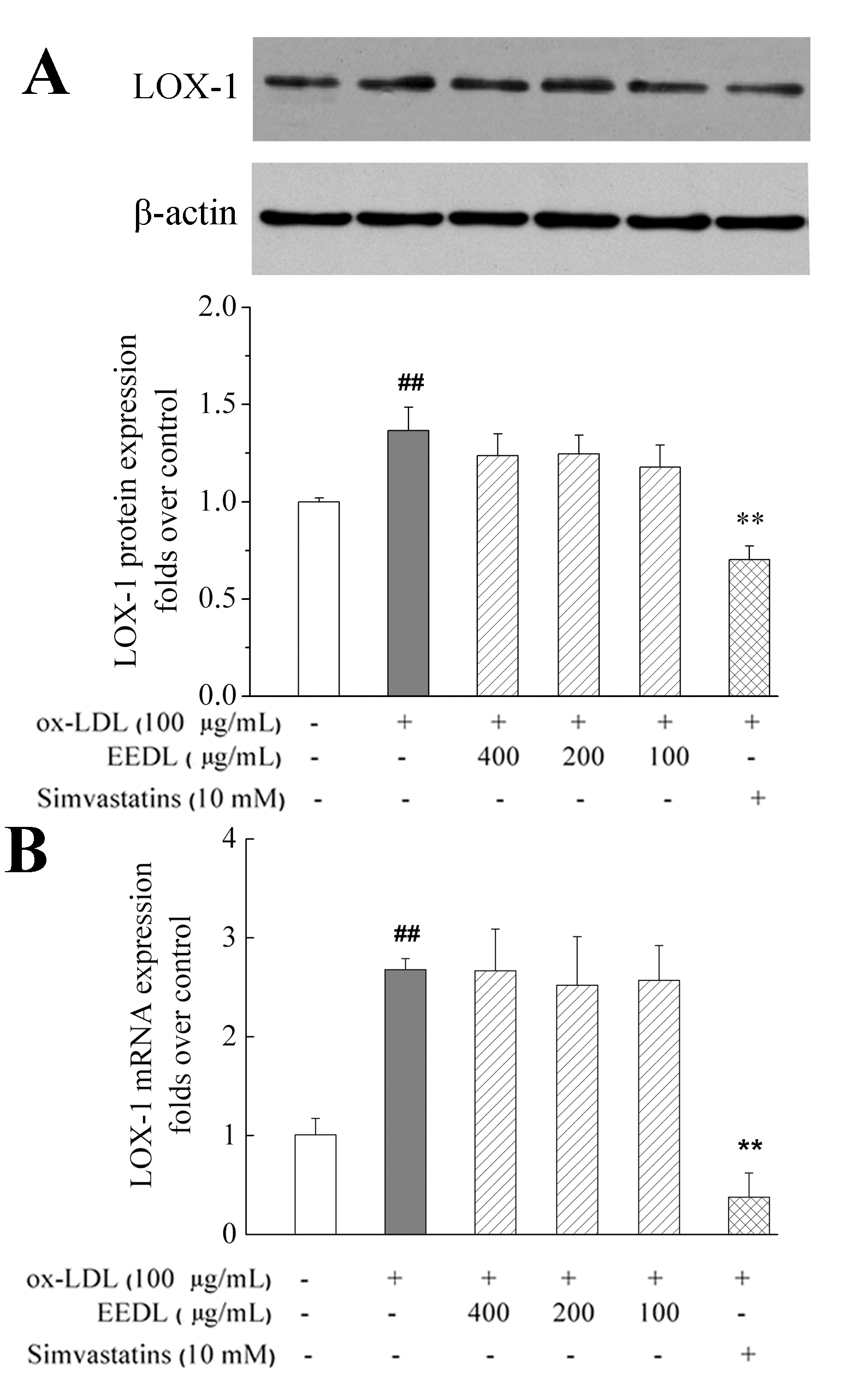

Supplement: FIGURE S2 — Effect of the EEDL on LOX-1 expression in ox-LDL-induced RAW 264.7 cells. (A) The protein expression of LOX-1 was detected using Western blotting. The relative optical density was quantified using NIH ImageJ software. Values are means ± SD (n = 3). (B) The mRNA expression of LOX-1 was detected using real-time RT-PCR. Values are means ± SD (n = 3) from three independent experiments. Significance compared with the control group or ox-LDL treated alone, ##P < 0.01 vs. the control group, ∗∗P < 0.01 vs. the ox-LDL-treated group. [file Image_2.TIF]
